# Delayed Diagnosis of Infective Endocarditis—Analysis of an Endocarditis Network

**DOI:** 10.3390/jcm15030924

**Published:** 2026-01-23

**Authors:** Shekhar Saha, Benjamin Zauner, Rainer Kaiser, Konstantinos Rizas, Martin Orban, Steffen Massberg, Sven Peterss, Christian Hagl, Dominik Joskowiak

**Affiliations:** 1 Department of Cardiac Surgery, Ludwig Maximillian University of Munich, 80539 Munich, Germany; 2 Department of Cardiology, Ludwig Maximillian University of Munich, 80539 Munich, Germany; 3 German Centre for Cardiovascular Research (DZHK), Partner Site Munich Heart Alliance, 80802 Munich, Germany; 4 University Aortic Centre Munich (LMU), Department of Cardiac Surgery, LMU University Hospital, 81377 Munich, Germany

**Keywords:** infective endocarditis, diagnosis

## Abstract

**Objectives**: The diagnosis of infective endocarditis (IE) is clinically challenging. This study aimed to examine an endocarditis network and the effects of delayed diagnosis. **Methods**: We reviewed the patients who were admitted for infective endocarditis at our institution between January 2012 and December 2021. Infective endocarditis was diagnosed according to ESC/EACTS guidelines for the management of endocarditis. Details of admitting hospitals were obtained from the German Hospital Directory. Data are presented as medians (25th–75th quartiles) or absolute values (percentages) unless otherwise specified. **Results**: A total of 812 consecutive patients were admitted to our centre for IE. Exact records on the time to diagnosis were available for 707 patients (87.1%). The patients were divided into two groups based on the time to diagnosis, i.e., up to 7 days (n = 509; 72.0% group ED) and more than 7 days (n = 198; 28.0% group LD). The EuroSCORE II (*p* = 0.001) and the EndoSCORE (*p* = 0.019) were significantly higher in the LD group. The median time to diagnosis was shorter in university hospitals as compared to non-teaching hospitals (*p* = 0.008) and among patients admitted to cardiology and cardiac surgery departments (*p* < 0.001). Patients diagnosed later had higher rates of tracheostomy (*p* < 0.001), longer ICU (*p* = 0.004) and hospital stays (*p* < 0.001) and higher in-hospital mortality (*p* = 0.027). We found that a delayed diagnosis (*p* = 0.040), stroke (*p* = 0.004), age > 75 years (*p* = 0.044) and atrial fibrillation (*p* < 0.001) were independently associated with in-hospital mortality. Furthermore, survival at 1 and 5 years was significantly higher in the ED group (*p* < 0.001). **Conclusions**: The diagnosis of IE may be influenced by a multitude of factors. Our results indicate that a delayed diagnosis is independently associated with an increased rate of in-hospital mortality. According to our results, an early diagnosis of IE may be associated with improved outcomes.

## 1. Introduction

Current guidelines recommend an interdisciplinary specialised approach for the diagnosis and management of IE [1]. This is reflected in clinical practice not only through the formation of specialised “endocarditis teams” but also through the formation of an endocarditis network of referring physicians and hospitals. The formation of endocarditis teams and endocarditis referral networks has been reported to result in the earlier referral of patients with fewer preoperative endocarditis-related complications [2,3].

The annual incidence of IE was reported to be 13.8 cases per 100,000 person-years in 2019, and the global burden of IE grew to over 1 million in 2019 [1,4]. The diagnosis of infective endocarditis (IE) is clinically challenging. Along with the rise in number of cases of IE, especially in older populations, multimorbidity makes the prompt diagnosis of IE challenging [5]. The exact point of beginning of symptoms is variable and may also depend on the patient, presence of comorbidities, course of the disease and pathogen [6,7]. Moreover, healthcare-associated IE accounts for 20–30% of all IE cases [4]. Blood-culture-negative endocarditis, prosthetic valve endocarditis, the specificity, sensitivity and availability of diagnostic methods, and atypical pathogens may add to the diagnostic challenge [1,8]. This study aims to examine an endocarditis network and the effects of delayed diagnosis.

## 2. Patients and Methods

### 2.1. Study Design

We reviewed the patients who were admitted for infective endocarditis at our institution between January 2012 and December 2021. Treatment and data acquisition were performed as part of routine patient care. This study was approved by the ethics board (No. 19-730 and 20-821), and the requirement to obtain patient consent was waived for this retrospective study. Infective endocarditis was diagnosed according to ESC/EACTS guidelines for the management of endocarditis [1,9]. The modified Duke criteria were used for clinical evaluation. In this study, time to diagnosis was defined as the time interval between admission at any hospital and determination of the diagnosis of infective endocarditis through clinical presentation or echocardiographic findings. We obtained details of admitting hospitals from the German Hospital Directory [10]. To predict the risk of mortality, the European System for Cardiac Operative Risk Evaluation II (EuroSCORE II) as proposed by Nashef et al. [11] and the EndoSCORE as proposed by Di Mauro et al. [12] were calculated. Cardiogenic shock was defined as persistent mean arterial pressure of less than 65 mmHg despite inotropic support [13]. Vascular and immunological phenomena were diagnosed as described in the 2023 ESC Guidelines for the management of endocarditis [1]. Data acquisition was based on institutional databases, and data were then de-identified. We analysed the characteristics, individual risk scores, and outcomes of these patients. The primary outcome was in-hospital mortality. The secondary outcome was long-term survival.

### 2.2. Data Collection, Statistical Analysis and Illustrations

Data were analysed using IBM SPSS version 25 (Statistical Package for the Social Sciences) (IBM-SPSS Inc., Armonk, NY, USA). Categorical variables were evaluated using the Chi-Squared and Fisher‘s exact methods, and continuous variables were evaluated using the Mann–Whitney U test. We used single imputation to replace missing values. Missing continuous values were replaced with the mean value for normally distributed variables and with the median value for non-normally distributed variables. Missing categorical values were replaced with the mode [14]. Survival analysis was performed with the Kaplan–Meier curve and log-rank test. Multivariate analysis incorporated binary logistic regression using a forward stepwise (conditional) model, where significance for entry was set at *p* < 0.05, and significance for exit was *p* < 0.10. The regression model was verified using the regression diagnostics as presented by Hickey et al. [15], which include a goodness of fit test as well as tests for autocorrelation, multicollinearity and heteroscedasticity. Data are presented as medians (25th–75th quartiles) or absolute values (percentages) unless otherwise specified. Illustrations were prepared using GraphPad Prism 10 (GraphPad Software Inc., Boston, MA, USA).

## 3. Results

### 3.1. Endocarditis Network

**Figure 1:**
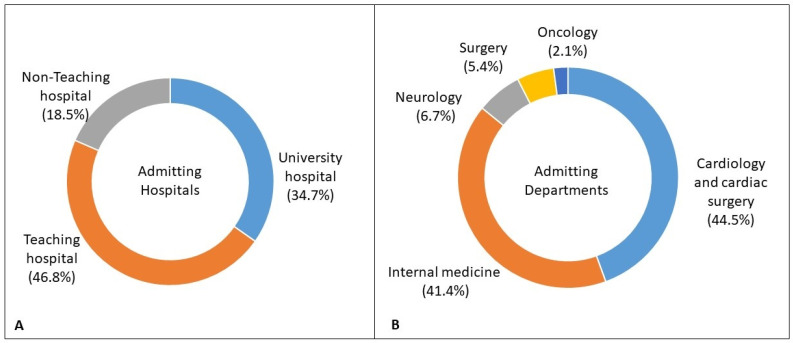
(**A**,**B**): Initial admitting departments and hospitals.

**Figure 2:**
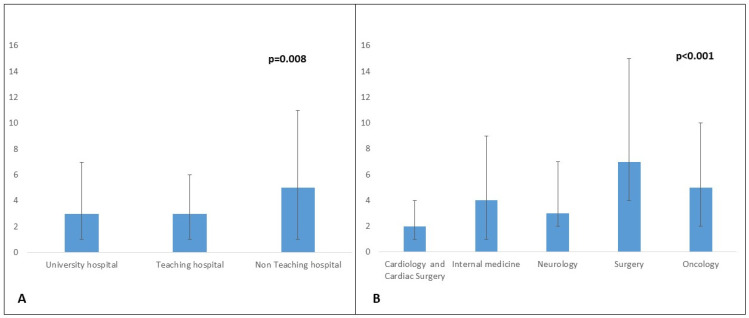
(**A**,**B**): Time to diagnosis.

An analysis of patient records revealed that patients were referred to our centre from 93 other hospitals. Details of the initial admitting departments and hospitals are provided in Figure 1A,B. Among the referral hospitals, echocardiography was ubiquitous. Following this, the most commonly available diagnostic tool was computer tomography (70 (75.3%)). Specialised diagnostic methods such as magnetic resonance imaging (48 (51.6%)) and 18F-fluorodeoxyglucose positron emission tomography (PET-CT) (21 (22.6%)) were not readily available. Details on the time to diagnosis are presented in Figure 2A,B. The median time to diagnosis was shorter in university hospitals as compared to non-teaching hospitals (*p* = 0.008) and among patients admitted to cardiology and cardiac surgery departments (*p* < 0.001).

### 3.2. Patient Population

Between January 2012 and December 2021, 812 consecutive patients were admitted to our centre for IE (Supplementary Table S1). Exact records on the time to diagnosis were available for 707 patients (87.1%). The patients were divided into two groups based on the time to diagnosis, i.e., up to 7 days (n = 509; 72.0% group ED) and more than 7 days (n =198; 28.0% group LD). Demographic characteristics are presented in Table 1. The median age was higher in group LD (*p* < 0.001). The EuroSCORE II (*p* = 0.001) and the EndoSCORE (*p* = 0.019) were significantly higher in the LD group. We found higher rates of hyperlipoproteinemia (*p* < 0.001), coronary artery disease (*p* = 0.013), new myocardial infarction (*p* = 0.004), atrial fibrillation (*p* = 0.039) and chronic obstructive pulmonary disease (*p* < 0.001) in the LD group. The ED group had a significantly higher rate of People Who inject Drugs (PWiD) (*p* = 0.037).

Details on endocarditis and its presentation are listed in Table 2. The median time to diagnosis was 1 day (1–3 days) in group ED and 12 days (8–18 days) in group LD (*p* < 0.001). Patients in group ED had higher rates of septic cerebral embolisms (*p* = 0.044). Although there was no difference in the rates of ICU admission between the groups (*p* = 0.205), patients in group LD had higher rates of ventilation on admission (*p* = 0.006) and cardiogenic shock (*p* = 0.032). A higher number of patients in group ED underwent surgery for IE (*p* < 0.001). The EuroSCORE II (*p* = 0.001) and the EndoSCORE (*p* = 0.019) were higher in the LD group.

Although we did not observe any differences in the rates of ICU admission (*p* = 0.205) between the groups, we observed a higher rate of ventilation on admission in the LD group (*p* = 0.006).

The details of IE are presented in Table 2. A significantly higher number of patients in the ED group underwent surgical treatment (*p* < 0.001), whereas a significantly higher number of patients in the LD group did not meet the Duke criteria (*p* = 0.023). There was no difference in the number of patients suffering from PVE, TAVR endocarditis, or double- or triple-valve endocarditis. Staphylococcal IE was diagnosed later more often (*p* = 0.004), whereas Streptococcal IE was diagnosed earlier more often (*p* < 0.001). Data on the diagnostics performed are presented in Table 3. About two-thirds of patients underwent computer tomography, whereas about 10% underwent PET-CTs. We found no difference with regard to the frequency of diagnostic modality between the groups. Patients with pulmonary hypertension were diagnosed later more often (*p* = 0.001), whereas patients with larger vegetations were diagnosed earlier (*p* = 0.005). LVEF, abscess formation and the rate of paravalvular leakage were similar between the groups.

### 3.3. Morbidities and Outcomes

**Figure 3:**
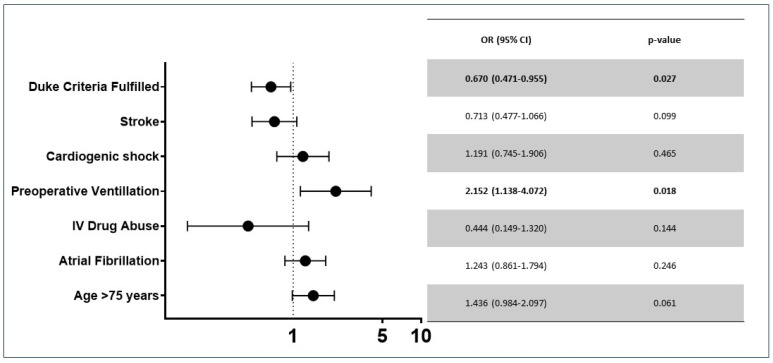
Forrest plot showing factors independently associated with delayed diagnosis.

**Figure 4:**
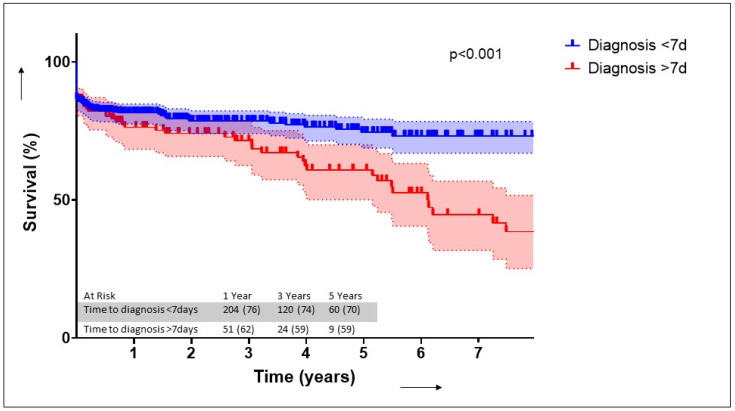
Kaplan–Meier survival curve.

Morbidities and outcomes are listed in Table 4. Patients diagnosed later had higher rates of tracheostomy (*p* < 0.001), longer ICU (*p* = 0.004) and hospital stays (*p* < 0.001) and higher in-hospital mortality (*p* = 0.027). There were no differences observed with regard to renal replacement therapy, ECLS support, IABP support, septic shock or pacemaker implantation. The in-hospital mortality was significantly higher in patients in the LD group (68 (13.4%) vs. 40 (20.2%); *p* = 0.027). We found that fulfilment of Duke criteria (OR 0.670 (0.471–0.955) *p* = 0.027) and preoperative ventilation (OR 2.152 (1.132–4.072) *p* = 0.018) were independently associated with a delayed diagnosis (Figure 3). We found that a delayed diagnosis (OR 1.72 (1.02–2.89), *p* = 0.040), stroke (2.07 (1.27–3.40), *p* = 0.004), age > 75 years (OR 1.72 (1.01–2.92) *p* = 0.044) and atrial fibrillation (OR 2.30 (1.40–3.77) *p* < 0.001) were independently associated with in-hospital mortality (Table 5). Survival at 1 (76% vs. 62%) and 5 years (71% vs. 59%) was significantly higher in the ED group (*p* < 0.001) (Figure 4).

## 4. Discussion

The diagnosis of IE is complex and may be challenging due to several factors, especially when it does not present with classic Oslerian manifestations [1,6]. Errors in diagnosis of IE have been reported to be as high as 54% [16]. Delayed diagnosis of IE in low- and middle-income countries has been reported to be associated with higher rates of complications such as congestive heart failure and persistent fever, and with a higher mortality rate in comparison to high-income countries [17]. The aetiology, presentation and causative pathogens of IE may be influenced by regional variation, changing prevalences of predisposing cardiac conditions, aging of the population, changes in PWiD, and increased exposure to intensive and invasive medical care [1,18]. Our results indicated that a delay in the diagnosis of infective endocarditis may result in higher rates of in-hospital mortality and poorer mid-term survival rates. We believe that the “endocarditis team” approach should be widened to incorporate the “endocarditis network”, to facilitate earlier diagnosis and optimal treatment of patients suffering from IE. This should include not only specialised tertiary care centres but also primary and secondary care centres. Furthermore, an endocarditis network facilitates increased awareness among referring physicians, which may lead to the rapid diagnosis and timely local management of patients with IE [2].

The time interval between the first onset of symptoms and diagnosis of IE has been reported to be related to the clinical presentation, patient characteristics and causative microorganisms [19]. When IE is caused by relatively avirulent microorganisms, such as *Streptococcus bovis* or HACEK (Haemophilus species, *Actinobacillus actinomycetemcomitans*, *Cardiobacterium hominis*, *Eikenella* species and *Kingella kingae*), the disease may present with an indolent clinical course, and patients gradually develop symptoms; it may take many months before a diagnosis is made [20]. When IE is caused by more virulent organisms such as Staphylococcus aureus, the course of the disease may be more aggressive, and a diagnosis is made earlier [20,21]. Other infections such as swine flu and SARS-CoV-2 infections may mask the presentation of IE, causing delays in diagnosis [22,23,24]. In our cohort, we found that Streptococcal IE was diagnosed earlier more frequently, whereas Staphylococcal IE was diagnosed later.

We found that the time to diagnosis depended on the admitting hospital and department. Patients being admitted to university hospitals and teaching hospitals were diagnosed quicker as compared to patients being admitted to non-teaching hospitals. Additionally, we found a significantly shorter time to diagnosis among patients being admitted to cardiology or cardiac surgery departments as compared to those being admitted to general surgery or oncology departments. Furthermore, specialised diagnostic approaches such as [18F]FDG-PET/CT and MRIs may not be readily available in the setting of primary and secondary care centres. In our network, echocardiography was ubiquitously available, whereas specialised imaging modalities such MRI and PET-CT were available in about half and one-fourth of the referral centres. Interestingly, we found no difference in the frequency of diagnostic modalities between the groups, i.e., with regard to time to diagnosis. A higher number of patients presenting with cardiogenic shock and/or ventilated on admission were diagnosed later. It has been reported that 90% of indications for ICU admittance include congestive heart failure, septic shock and neurological complications [25]. It is important to consider IE as a differential diagnosis among these patients and to initiate appropriate treatment. Thus, despite improvements in diagnostic criteria and diagnostic tools, the diagnosis of IE requires a high level of clinical suspicion, physician competence and awareness, and interdisciplinary management.

Although the Duke criteria have undergone modifications over the years, the modified 2023 Duke criteria have been reported to have a sensitivity of 69% [26]. In our cohort, we found that fulfilment of the Duke criteria was independently associated with an earlier diagnosis of IE. The use of transthoracic (TTE) and transoesophageal (TOE) ultrasound was found to be ubiquitous in our referral network. TTE has been reported to have a sensitivity of up to 98%. However, this may be limited in cases of PVE [1,6]. The presence of vegetations has been reported to have a sensitivity of up to 60% [6]. We found that a significantly higher number of patients with vegetations >10 mm were diagnosed earlier. As mentioned above, patients presenting with classical manifestations such as positive blood cultures, cerebral embolisms and vascular phenomena were diagnosed earlier. Brain and whole-body imaging with computed tomography, [18F]FDG-PET/CT and/or magnetic resonance imaging have an IB recommendation in symptomatic NVE and PVE patients [1]. However, in our endocarditis network about three-fourths of the hospitals had access to computed tomography, half to MRI and less than one-fourth to [18F]FDG-PET/CT. An even smaller number of patients underwent specialised diagnostic tests such as [18F]FDG-PET/CT and MRI (Table 3). Although guidelines govern clinical practice, it is widely known that implementation of guideline recommendations varies based on local resource settings at the centre, region and country level [27]. Furthermore, not all health care facilities may have access to specialised diagnostic modalities such as [18F]FDG-PET/CT and Single-Photon Emission Computed Tomography (SPECT). In low-resource settings, priority should be given to increased awareness, an endocarditis team approach, early diagnosis and treatment, prompt surgical treatment and reducing the recurrence of infective endocarditis [27].

Patients with prosthetic heart valves are the classical at-risk patients for IE. Despite this, more than 25% of the PVE cases required more than 7 days to be diagnosed. Although we observed no significant difference between the groups, this delay in the diagnosis of PVE is alarming. Another interesting sub-group is those patients suffering from TAVR IE. Although such cases were rare, about one in four cases of patients suffering from TAVR IE were diagnosed later. With the exponentially rising number of TAVR implantations worldwide, especially in lower risk categories, TAVR IE is a significant clinical challenge [28].

The demographics of patients suffering from IE are changing [18]. Infective endocarditis (IE) has a different clinical profile in elderly patients, who have higher rates of nosocomial infections, intracardiac prosthetic devices and mitral valve endocarditis. Furthermore, elderly patients may present with atypical symptoms such as lethargy, fatigue, malaise, anorexia, weight loss and stroke, which may be attributed to aging, malignancies or other disorders, thereby delaying diagnosis and treatment [29,30]. In our cohort, we too found that patients diagnosed later were significantly older than those receiving a prompt diagnosis. There were no differences in the valve affected with regard to the time to diagnosis. Furthermore, we found that there was no difference in the number of patients with previous endocarditis episodes between the groups. Rates of recurrence (inclusive of relapses and reinfections) have been reported to range within 2–9% among IE survivors.

Although only about half the patients suffering from IE require surgery, it has been reported that patients treated conservatively despite indications for surgery have poorer outcomes [1,31]. Urgent surgery in cases of left-sided NVE or PVE with severe valvular regurgitation, oedema or cardiogenic shock, heart failure and uncontrolled infection has a class IB recommendation in the current guidelines [1]. Early surgery in patients with IE and large vegetations significantly lowers the risk of mortality from any cause and embolic events by effectively decreasing the risk of systemic embolism [32]. Therefore, delays in diagnosis may lead to a delay in surgery, which is associated with an increased risk of local disease progression, the development of abscesses and pronounced tissue destruction, sepsis, multiple organ failure, embolic events and poor prognosis [1,2,31]. Our results indicate that a significantly higher number of patients underwent surgery when diagnosed earlier.

Our study indicates that the delay in diagnosing IE can be multifactorial. Among older patients, typical presenting symptoms such as fever and malaise may be attributed to chronological age. Furthermore, multimorbidity and other presenting symptoms such as cardiogenic shock, sepsis and stroke may delay the diagnosis. Despite modern diagnostic methods such as MRI PET and SPECT, the mainstay for the diagnosis of IE remains blood cultures. In cases of hard-to-detect pathogens, such as *Cutibacterium acnes*, *Bartonella* sp., *Tropheryma whipplei* or fungal species, the diagnosis may be delayed. Additionally, although super-specialisation does result in optimal specialty-specific outcomes, it should be kept in mind that IE may affect all patients. Along with prolonged hospital and ICU stays, a delayed diagnosis was associated with a significantly higher rate of mortality. Our results indicate that a delayed diagnosis is independently associated with an increased rate of in-hospital mortality. On follow-up we found that patients diagnosed earlier had higher rates of survival as compared to those diagnosed later. All of this suggests that an early diagnosis of IE may be associated with improved outcomes.

### Limitations

This was a retrospective single-centre study with the inherent limitations of such an analysis, including lack of generalisability to other regions or healthcare systems. The small number of patients is associated with low power in statistical analyses. Patients who remained undiagnosed or transferred to other tertiary care centres were outside of the scope of this study.

## 5. Conclusions

The diagnosis of IE may be influenced by a multitude of factors. With the ever-changing demographic of patients suffering from IE, a high level of physician awareness and competence is required to ensure timely diagnosis. The timely diagnosis of IE is of paramount importance to initiate pathogen-specific antibiotic treatment, to halt progression of the disease and embolisation, to lower complication rates and to achieve optimal outcomes.

## Figures and Tables

**Table 1:** Baseline parameters. Data are presented as medians (25th–75th percentiles) or absolute numbers (percentages). BMI: body mass index; COPD: chronic obstructive pulmonary disease; EuroSCORE II: European System for Cardiac Operative Risk Evaluation II; HIV: human immunodeficiency virus; ICU: intensive care unit; NYHA: New York Heart Association; PCI/PTCA: percutaneous coronary intervention/percutaneous transluminal coronary angioplasty; PWiD: People Who inject Drugs.

	Time to Diagnosis <7 Days (n = 509)	Time to Diagnosis >7 Days (n = 198)	*p*-Value
Age (years)	66 (53–74)	68 (61–76)	**<0.001**
Male	365 (71.7)	142 (71.7)	1.000
BMI (kg/m^2^)	25.0 (22.9–27.7)	25.2 (23.7–28.0)	0.386
NYHA Class			**0.019**
Class I	25 (4.9)	5 (2.5)	
Class II	154 (30.4)	42 (21.2)	
Class III	285 (56.2)	124 (62.6)	
Class IV	45 (8.9)	27 (13.6)	
EuroSCORE II	4.6 (2.3–10.0)	7.4 (3.6–16.8)	**0.001**
EndoSCORE	11.4 (6.9–18.1)	12.3 (6.9–24.2)	**0.019**
Comorbidities			
Arterial Hypertension	370 (72.7)	154 (77.8)	0.181
Insulin-Dependent Diabetes Mellitus	50 (9.8)	28 (14.1)	0.109
Hyperlipoproteinemia	203 (39.9)	108 (54.5)	**<0.001**
Coronary Artery Disease			**0.013**
Single-Vessel Disease	46 (9.0)	32 (16.2)	
Two-Vessel Disease	39 (7.7)	17 (8.6)	
Three-Vessel Disease	5 (10.8)	28 (14.1)	
Peripheral Artery Disease	43 (8.4)	23 (11.6)	0.197
Atrial Fibrillation	151 (30.1)	76 (38.4)	**0.039**
Pacemaker	61 (12.0)	28 (14.1)	0.450
PCI/PTCA	41 (8.1)	41 (20.7)	**<0.001**
New Myocardial Infarction	19 (3.7)	19 (9.6)	**0.004**
Chronic Kidney Disease	101 (19.8)	52 (26.3)	0.068
Dialysis	23 (4.5)	8 (4.0)	1.000
Immunosuppressive Therapy	41 (8.1)	21 (10.6)	0.301
Malignancy	124 (24.4)	50 (25.3)	0.846
Alcohol Abuse (%)	46 (9.0)	22 (11.1)	0.397
PWiD	27 (5.3)	4 (2.0)	**0.037**
HIV Infection	3 (0.6)	3 (1.5)	0.357
Smoker (%)	85 (16.7)	28 (14.1)	0.426
COPD (%)	38 (7.5)	35 (17.7)	**<0.001**
Previous Cardiac Surgery (%)	145 (28.5)	61 (30.8)	0.580
Clinical Presentation			
Previous Endocarditis (%)	27 (5.3)	6 (3.0)	0.237
Fever (%)	289 (56.8)	105 (53.0)	0.399
Septic Cerebral Embolism (%)	147 (28.9)	44 (22.2)	**0.044**
ICU Admission (%)	151 (29.7)	69 (34.8)	0.205
Ventilation on Admission (%)	33 (6.5)	26 (13.1)	**0.006**
Cardiogenic Shock (%)	90 (17.7)	48 (24.2)	**0.032**

Bold is only for *p* values < 0.05.

**Table 2:** Endocarditis-specific data. Data are presented as absolute numbers (percentages). BCNIE: blood-culture-negative infective endocarditis; CoNS: coagulase-negative Staphylococci; HACEK: Haemophilus, Aggregatibacter, Cardiobacterium, Eikenella, Kingella; TAVR: Transcatheter Aortic Valve Replacement.

	Time to Diagnosis <7 Days (n = 509)	Time to Diagnosis >7 Days (n = 198)	*p*-Value
Time to diagnosis (days)	1 (1–3)	12 (8–18)	<0.001
Surgical treatment	366 (71.9)	102 (51.5)	<0.001
Valves affected			
Prosthetic valve endocarditis	143 (28.1)	52 (26.3)	0.641
TAVR endocarditis	29 (5.9)	9 (4.5)	0.710
Mitral valve	227 (44.6)	92 (46.5)	0.674
Aortic valve	318 (62.5)	119 (60.1)	0.605
Tricuspid valve	32 (6.3)	6 (3.0)	0.096
Pulmonary valve	1 (0.2)	1 (0.5)	0.482
Double-valve endocarditis	70 (13.8)	20 (10.1)	0.210
Triple-valve endocarditis	2 (0.4)	0 (0.0)	1.000
Modified Duke criteria			
Major criteria			
Positive blood cultures	332 (65.2)	112 (56.6)	0.037
Echocardiographic evidence	461 (90.6)	174 (87.9)	0.332
Minor criteria			
Predisposing heart condition or intravenous drug use	243 (47.7)	83 (41.9)	0.179
Fever	289 (56.8)	105 (53.0)	0.399
Vascular phenomena	197 (38.7)	54 (27.3)	0.005
Immunologic phenomena	20 (3.9)	4 (2.0)	0.253
Positive blood culture not meeting major criterion (%)	77 (15.1)	54 (27.3)	<0.001
Patients not meeting Duke criteria (%)	169 (33.2)	84 (42.4)	0.023
Pathogens			
BCNIE (%)	80 (15.7)	29 (14.6)	0.817
Gram-positive pathogens (%)	406 (79.8)	156 (78.8)	0.757
Staphylococci	169 (33.2)	89 (44.9)	0.004
CoNS (%)	45 (8.8)	22 (11.1)	0.391
S. aureus (%)	126 (24.8)	68 (34.3)	0.011
Streptococci (%)	153 (30.1)	34 (17.2)	<0.001
Enterococci (%)	66 (13.0)	28 (14.1)	0.712
Gram-negative pathogens (%)	24 (4.7)	13 (6.6)	0.348
HACEK pathogens (%)	9 (1.8)	1 (0.5)	0.298

**Table 3:** Diagnostics: Data are presented as absolute numbers (percentages). CT: computer tomography; LVEF: left ventricular ejection fraction; MRT: magnetic resonance tomography; PET CT: positron emission tomography–computed tomography.

	Time to Diagnosis <7 Days (n = 509)	Time to Diagnosis >7 Days (n = 198)	*p*-Value
Diagnostics			
Transoesophageal Echocardiography	475 (93.3)	190 (96.0)	0.217
Chest CT	336 (66.0)	126 (63.6)	0.598
Cerebral CT	317 (62.3)	122 (61.6)	0.931
Abdominal CT	168 (33.0)	74 (37.4)	0.290
PET CT	48 (9.4)	26 (13.1)	0.171
Cardiac MRI	6 (1.2)	1 (0.5)	0.680
Cerebral MRI	111 (21.8)	44 (22.2)	0.920
Echocardiography			
LVEF			0.057
>50%	410 (80.6)	144 (72.7)	
30–50%	85 (16.7)	44 (22.2)	
<30%	14 (2.8)	10 (5.1)	
Pulmonary Hypertension	86 (16.9)	56 (28.3)	0.001
Vegetation	450 (88.4)	176 (88.9)	0.896
Size of Vegetation			0.005
<5 mm	41 (8.1)	20 (10.1)	
5–10 mm	168 (33.0)	89 (44.9)	
>10 mm	241 (47.3)	66 (33.3)	
Abscess	111 (21.8)	36 (18.2)	0.304
Paravalvular Leakage	57 (11.2)	15 (7.6)	0.168

**Table 4:** Morbidities and outcomes. Data are presented as absolute numbers (percentages). ECMO: extracorporeal membrane oxygenation; IABP: intra-aortic balloon pump; ICU: intensive care unit.

	Time to Diagnosis <7 Days (n = 509)	Time to Diagnosis >7 Days (n = 198)	*p*-Value
Morbidities			
Renal replacement therapy	56 (13.7)	25 (18.8)	0.163
ECLS support	26 (6.4)	8 (6.0)	1.000
IABP support	7 (1.7)	4 (3.0)	0.478
Septic shock	67 (16.5)	30 (22.4)	0.122
Pacemaker implantation	37 (9.1)	11 (8.2)	0.862
Tracheostomy	15 (3.7)	20 (14.9)	<0.001
Outcomes			
Hospital stay (days)	22 (14–35)	25 (15–39)	<0.001
Ventilation time	16 (8–48)	24 (8–128)	0.109
ICU stay	4 (2–8)	6 (3–13)	0.004
In-hospital mortality	68 (13.4)	40 (20.2)	0.027
Discharge			
Cardiac rehabilitation centre	168 (33.0)	49 (24.7)	0.037
Hospital	161 (31.6)	65 (32.8)	0.788
Home	112 (22.0)	44 (22.2)	1.000

**Table 5:** Multivariable analysis of factors independently associated with in-hospital mortality. CAD: coronary artery disease; LVEF: left ventricular ejection fraction.

	OR (95%CI)	*p*-Value
Delayed diagnosis	1.72 (1.02–2.89)	0.040
Stroke	2.07 (1.27–3.40)	0.004
Age > 75 years	1.72 (1.01–2.92)	0.044
Atrial fibrillation	2.30 (1.40–3.77)	<0.001
LVEF < 30	1.48 (0.45–4.92)	0.519
CAD	1.01 (0.61–1.68)	0.972
Tracheostomy	0.443 (0.145–1.35)	0.152

## Data Availability

Data are not openly available due to national data safety regulations and are available from the corresponding author upon reasonable request.

## Supplementary Materials

The following supporting information can be downloaded at https://www.mdpi.com/article/10.3390/jcm15030924/s1, Table S1: Population overview.
