# Supplementary material for: Delayed Diagnosis of Infective Endocarditis—Analysis of an Endocarditis Network
Source: J Clin Med. 2026 Jan 23;15(3):924. doi: 10.3390/jcm15030924 (PMC12898327; doi:10.3390/jcm15030924)
Supplement: Supplementary file 1 [file jcm-15-00924-s001.zip › jcm-4067926-supplementary.pdf]

	(n=812)
Demographics	
Age (years)	67 (55-75)
Male	585 (72.0)
BMI (kg/m^2)	24.7 (14.0-36.0)
EuroSCORE II (%)	5.0 (2.4-10.1)
EndoSCORE (%)	11.4 (6.9-19.6)
Hospital stay (days)	23 (14-36)
In-hospital mortality (%)	124 (15.3)
Valves affected	
PVE (%)	234 (28.8)
Mitral valve (%)	354 (43.6)
Aortic valve (%)	514 (63.3)
Tricuspid valve (%)	43 (5.3)
Pulmonary valve (%)	3 (0.4)
Double valve IE (%)	103 (12.7)
Triple valve IE (%)	2 (0.2)
Previous cardiac surgery (%)	248 (30.5)
Co-morbidities	
Hypertension (%)	607 (74.8)
Hyperlipoproteinemia	362 (44.6)
Peripheral artery disease (%)	75 (%)
Atrial Fibrillation (%)	259 (32.2)
Pacemaker (%)	102 (12.6)
Chronic Kidney Disease (%)	180 (22.2)
Dialysis (%)	33 (4.1)
Immunosuppresive treatment (%)	67 (%)
Malignancy (%)	190 (23.4)
PWiD	33 (4.1)
HIV Infection (%)	7 (0.7)
COPD (%)	83 (10.2)
Cardiogenic Shock (%)	151 (18.6)
Endocarditis data	
BCNIE (%)	126 (15.5)
Gram Positive (%)	646 (79.6)
Staphylococcus sp.	287 (35.3)
S. aureus (%)	211 (26.0)
CoNS (%)	79 (9.7)
Streptococcus sp.	215 (26.6)
Enterococcus sp.	119 (14.7)
Gram Negative (%)	41 (5.0)
Duke Criteria Fulfilled (%)	515 (63.4)
Vegetations (%)	716 (88.2)
Abscess (%)	176 (21.7)
Paravalvular leak (%)	81 (10.0)
Previous IE (%)	24 (3.0)

Supplementary Table S1.: Population Overview. BCNIE: Blood Culture Negative Infective Endocarditis, COPD: Chronic Obstructive Pulmonary Disease, , HIV: Human Immunodeficiency Virus, IE : Infective Endocarditis, PVE : Prosthetic Valve Endocarditis, PWiD: People Who inject Drugs
